# New microvascular ultrasound techniques: abdominal applications

**DOI:** 10.1007/s11547-023-01679-6

**Published:** 2023-07-26

**Authors:** Roberto Cannella, Giulia Pilato, Mariasole Mazzola, Tommaso Vincenzo Bartolotta

**Affiliations:** Department of Biomedicine, Neuroscience and Advanced Diagnostics (BiND), University of Palermo, Via del Vespro 129, 90127 Palermo, Italy

**Keywords:** Superb microvascular imaging, Microvascular flow imaging, Micro-flow imaging, Contrast-enhanced ultrasound

## Abstract

Microvascular ultrasound (MVUS) is a new ultrasound technique that allows the detection of slow-velocity flow, providing the visualization of the blood flow in small vessels without the need of intravenous contrast agent administration. This technology has been integrated in the most recent ultrasound equipment and applied for the assessment of vascularization. Compared to conventional color Doppler and power Doppler imaging, MVUS provides higher capability to detect intralesional flow. A growing number of studies explored the potential applications in hepatobiliary, genitourinary, and vascular pathologies. Different flow patterns can be observed in hepatic and renal focal lesions providing information on tumor vascularity and improving the differential diagnosis. This article aims to provide a detailed review on the current evidences and applications of MVUS in abdominal imaging.

## Introduction

Evaluation of vascular flow is an important part of ultrasound examination. Conventional Doppler techniques, such as color Doppler imaging (CDI) and power Doppler imaging (PDI), are commonly used to estimate vascularization of focal lesions. However, CDI and PDI have limited sensibility for the detection of slow vascular flow. Microvascular ultrasound (MVUS) is a new ultrasound technique that allows the detection of slow-velocity flow, providing the visualization of the blood flow in small vessels without the need of intravenous contrast agent administration [1, 2]. Recently, MVUS techniques have been developed by different vendors and integrated in the ultrasound equipment for clinical use, as provided in Table 1.

**Table 1:** New microvascular ultrasound imaging techniques applied in abdominal pathologies

Commercial name	Abbreviation	Vendor
Microflow imaging	MFI	Philips Healthcare
Microvascular imaging	MVI	GE Healthcare
Microvascular flow imaging	MV-Flow™	Samsung Medison Co
Superb microvascular imaging	SMI	Canon Medical Systems

Detailed description of the MVUS has been provided in prior publications [3–5]. Briefly, CDI and PDI techniques eliminate artifacts caused by tissue movements and clutter by applying a monodimensional wall filter, which also removes the slow blood flow signals that occupy the same bandwidth on the frequency domain [4]. The microvascular technology applies an advanced filter to separate the slow flow signal from the clutter signal [5]. This allows to preserve the slow flow signal originating from microvasculature. The corresponding flow information can be displayed in color mode on conventional gray-scale ultrasound images with embedded color-encoded flow signals or as monochrome mode which focuses on the vascular signal only and further enhances the vascular pattern by suppressing the background signal [3]. The superb microvascular imaging allows to calculate the vascular index, which is a quantitative parameter representing the percentage of color pixels on the total pixels number within a region of interest [3, 5]. It can be calculated with a dedicated application of the US device by placing a region of interest with standardized size or by drawing the contour of the area of interest [5]. Finally, it should be noted that the application of MVUS in abdominal imaging can be limited by the lesion depth with microvascular flow being less detectable in deeper regions, smaller box size compared to other Doppler techniques, and large motion artifacts [1].

The MVUS have been widely applied for the diagnosis of thyroid pathologies and breast lesions [6, 7]. Recently, several abdominal applications have been explored to improve the diagnosis of focal and diffuse abdominal pathologies (Table 2). This article aims to provide an up-to-date review on the current evidences and applications of MVUS in abdominal imaging.

**Table 2:** Current results of microvascular ultrasound studies in abdominal organs

Organ	Applications	Key results
Liver	Differential diagnosis between focal liver lesions [9–16]	Improved detection of tumor vascularity. Hemangioma: strip rim pattern (26–48%) or nodular rim pattern (37–52%). FNH: spoke-wheel pattern (41–67%). HCC: non-specific or irregular pattern (48–93%)
	Assessment of treatment response [18]	Detection of residual intratumoral flow after transarterial chemoembolization
	Fibrosis staging [25–28]	Vascular changes can predict the severity of fibrosis
	Diagnosis of steatosis [29]	Vascular index inversely correlated with hepatic steatosis
	Assessment of liver transplant recipients [30–33]	Improved visibility of the hepatic artery and diagnosis of hepatic artery thrombosis
Gallbladder	Diagnosis of acute cholecystitis [34]	Superb microvascular imaging value predicted acute cholecystitis
	Diagnosis of gallbladder lesions [37]	Superb microvascular imaging may help in the differential diagnosis
Kidney	Differential diagnosis between renal lesions [41–45]	Improved detection of intratumoral vascularization. Malignant tumors: annular or ring-like flow pattern (65–67%)
	Diagnosis of acute pyelonephritis [46]	Improved detection and diagnosis
	Evaluation of renal function [47, 48]	Cortical flow and vascular index correlated with the severity of chronic kidney disease
Bladder	Acute cystitis [50]	Higher vascular index was associated with acute cystitis
	Vesicoureteral reflux [51]	Demonstrated reversed ureteral jet and/or renal pelvic swirl sign
Prostate	Diagnosis of prostate cancer [52, 53]	Higher vascularity in malignant lesions and lesions with higher Gleason score
Gynecological	Evaluation of ovarian vascularization [54]	Improved detection of ovarian vascularization
	Treatment of uterine fibroids [55]	Higher pretreatment vascularity was associated with higher volume reduction
	Pregnancy [56–63]	Assessment of placenta pathologies. Assessment of fetal organs vascularization
Vascular	Diagnosis of endoleak [66–68]	High accuracy for endoleak detection

## Hepatic applications

**Fig. 1:**
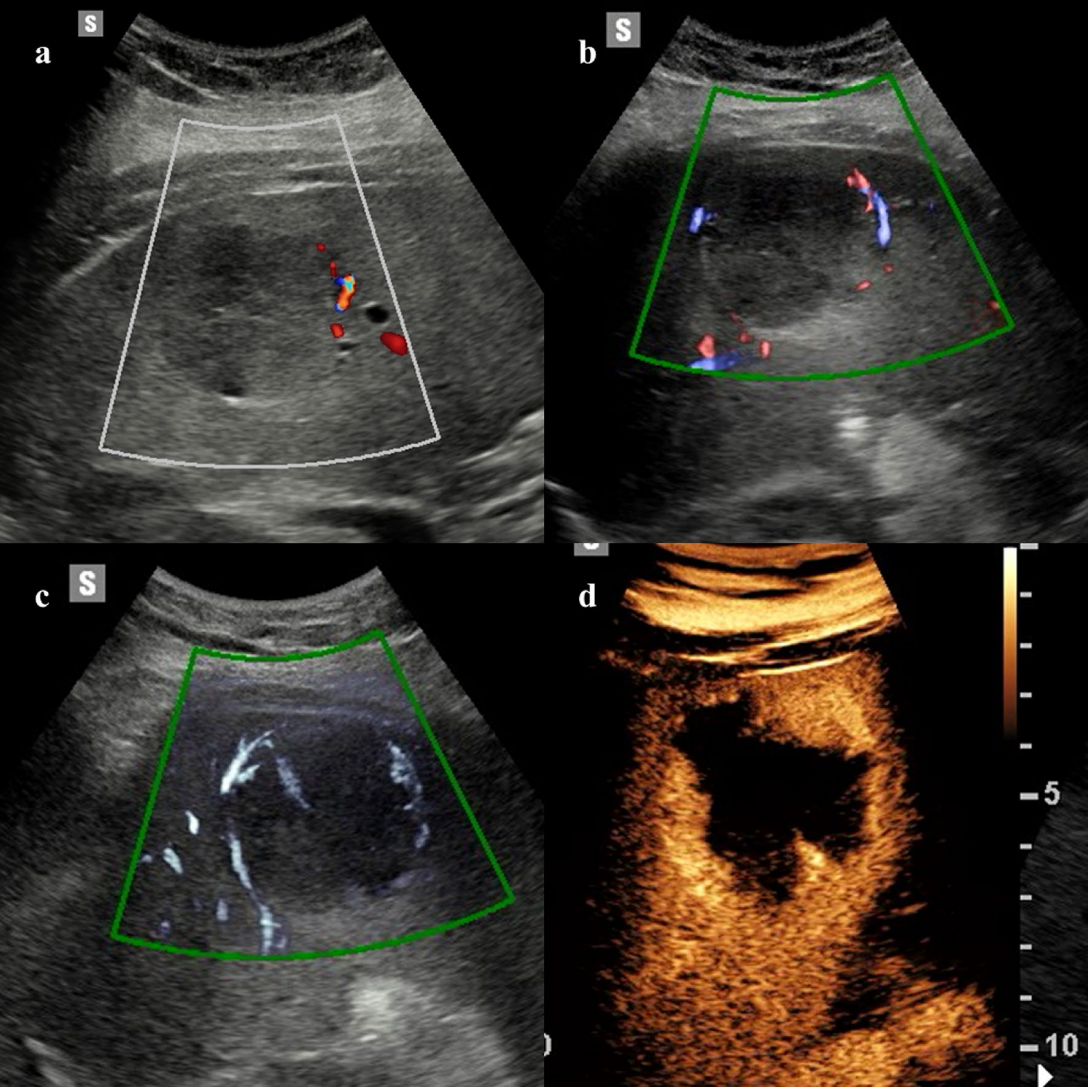
40-year-old woman with a 5.7 cm hepatic hemangioma incidentally detected on abdominal ultrasound examination. Color (**a**) and directional power Doppler (**b**) imaging demonstrate minimal peripheral vascularization. Microvascular flow imaging shows a strip rim pattern of vascularization (**c**). Contrast-enhanced ultrasound (CEUS) confirmed the diagnosis of hepatic hemangioma by showing the peripheral globular enhancement (**d**, 63 s after contrast injection)

**Fig. 2:**
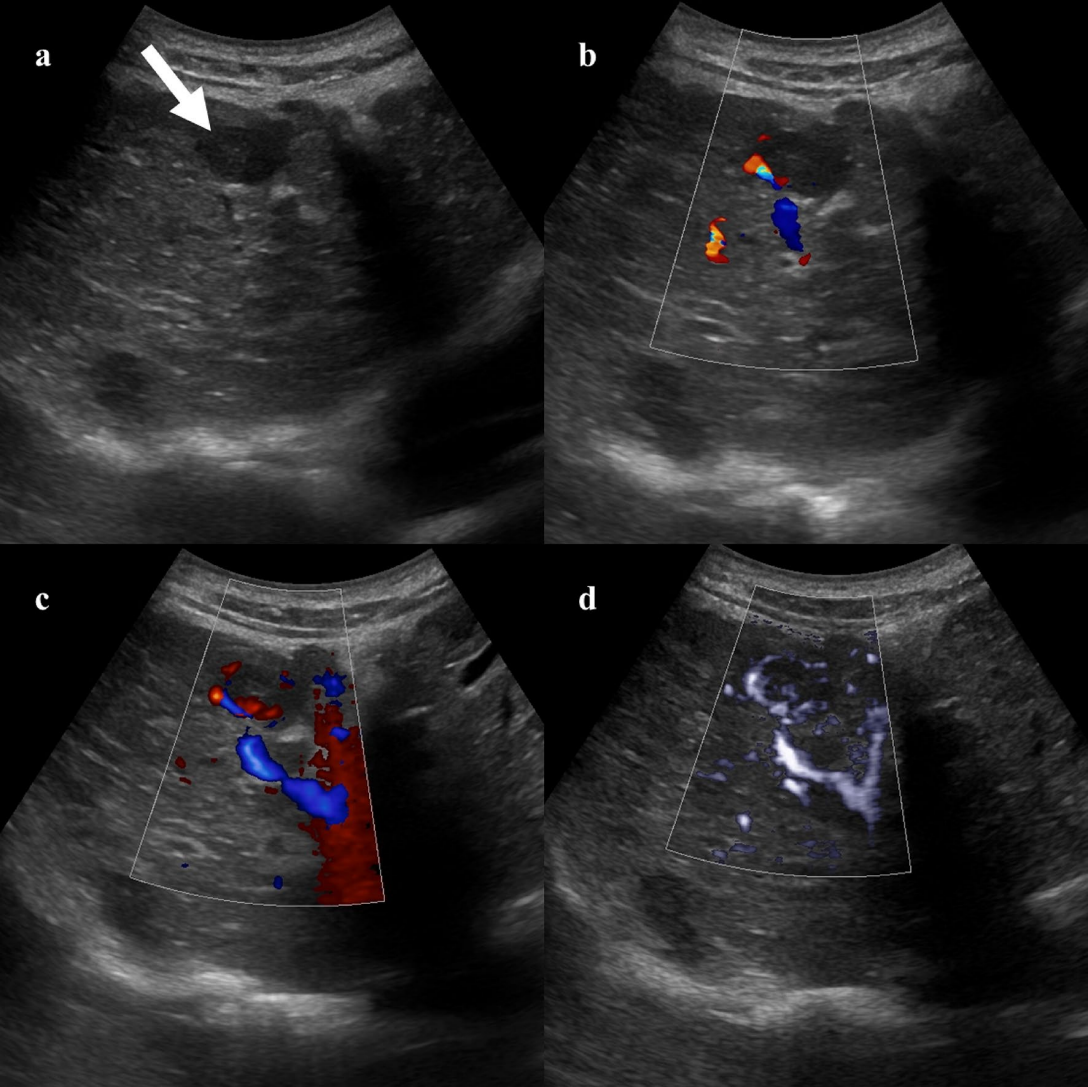
78-year-old man with hepatocellular carcinoma. B-mode ultrasound (**a**) shows a 2.0 cm hypoechoic lesion in the right hepatic lobe (arrow). Color (**b**) and power Doppler (**c**) imaging demonstrate mild peripheral vascularization. Microvascular imaging clearly depicts both peripheral and irregular intratumoral vascular pattern (**d**)

Vascularization of hepatic lesions with MVUS was analyzed by several studies, and different vascular patterns were associated with specific lesions [8, 9]. In an initial study on 29 liver lesions, Lee et al. [10] reported nodular rim pattern and spotty dot-like pattern in 33% and 20% of hemangiomas, respectively, while focal nodular hyperplasia (FNH) exhibited more commonly a spoke-wheel (43%) or radiating (29%) pattern. Dubinsky et al. [11] and Han et al. [12] identified a higher number of central and peripheral vessels in hepatocellular carcinomas (HCCs) compared to benign lesions by using MVUS. In a study including 92 hemangiomas, the most common vascular patterns detected by superb microvascular imaging were the strip rim (48.4%) and the peripheral nodular rim (37.1%) patterns (Fig. 1) [13]. However, about one-third of the hemangiomas did not show any vascular signal, more frequently being lesions smaller than 2 cm [13]. In a recent prospective study, Jeon and colleagues [14] demonstrated a significantly higher sensitivity for the vascular flow detection and higher tumor vascularity score with MVUS compared to CDI and PDI. In this study, hemangiomas demonstrated more commonly a nodular rim pattern (52.2%) or strip rim pattern (26.1%), and FNHs were associated with a spoke-wheel pattern (66.7%), while malignant tumors had an irregular nonspecific vascular pattern (66.7%) [14]. The irregular vascular pattern (Fig. 2) was the most commonly reported pattern in HCCs by Yang et al. [15], while Bae et al. [16] described the basket pattern in HCC characterized by a combination of peripheral rim of vascularization and irregular internal vessels. Recent studies reported a significant correlation between the quantification of vascular index and the microvessel density at the histopathological analysis in HCCs and hepatic metastases [15, 17]. Moreover, Kang et al. [18] prospectively evaluated 100 patients with HCC treated with transarterial chemoembolization. In this study microflow imaging had a sensitivity of 79.3% and a specificity of 80% for the detection of residual intratumoral flow [18].

The different vascular patterns may be explained by the different histopathological tumoral features. Hemangiomas were associated with a peripheral strip rim pattern or nodular rim pattern with dot-like spots, which can be related to the dilated peripheral vascular spaces. The spoke-wheel pattern of FNH is related to its internal vascular architecture, with intralesional arteries radiating from the center toward the periphery of the lesion [19, 20]. Peripheral draining veins can also be visualized in FNH [21]. The irregular vascular pattern of malignant tumors and HCCs reflects the abnormal tumoral neoangiogenesis, leading to the development of irregular intralesional vessels.

Ultrasound evaluation of the nonlesional liver parenchyma is also performed to assess morphological changes associated with advanced liver disease and detection of hepatic steatosis. Liver biopsy is the reference standard for the fibrosis staging, but it is an invasive procedure that carries risks of complications and sampling errors. Therefore, several advanced imaging methods have been evaluated over time as potential noninvasive tools for the fibrosis staging [22–24]. Vascular changes and distortions, as result of progressive fibrosis accumulation, were explored with MVUS [25, 26]. A study by Balik et al. [27] identified subcapsular small vessels blunting on superb microvascular imaging as a relevant finding for the prediction of hepatic fibrosis. In the study by Tosun and colleagues [28], the vascular score obtained with the superb microvascular imaging significantly correlated with the liver fibrosis stage at histopathology. Interestingly, in that study the vascular score predicted fibrosis with higher accuracy compared to the SWE [28].

Furthermore, Gao et al. [29] correlated the vascular index obtained with superb microvascular imaging with the presence of hepatic steatosis using the proton density fat fraction as reference standard. In that study, patients with hepatic steatosis had a significantly lower vascular index compared to normal controls, likely reflecting the changes in microvascularity due to fat accumulation within the hepatocytes [29].

MVUS was also employed to improve the assessment of the hepatic artery in posttransplant evaluation, showing improved visibility compared to CDI; therefore, MVUS could be used to improve the diagnosis of hepatic artery stenosis and thrombosis in transplanted patients [30–32]. In an initial experience by Jang and colleagues [30] on 56 patients, superb microvascular imaging improved the visibility of hepatic artery compared to CDI and provided a good-to-excellent inter-reader reproducibility for hepatic artery measurements. Similarly, Güven et al. [31] reported that superb microvascular imaging allowed to visualize vascular flow in the hepatic artery in 16/20 patients in which normal flow was undetectable on CDI. In pediatric liver transplantation, the hepatic artery visibility score was significantly higher in superb microvascular imaging compared to CDI [32]. In a study by Gu et al. [33], the MVUS provided a sensitivity of 100% and a specificity of 98.9% for the diagnosis of hepatic artery thrombosis in 105 transplanted children, which was similar to CDI (sensitivity of 100% and specificity of 92.8%).

## Gallbladder, pancreatic and splenic applications

### Gallbladder

**Fig. 3:**
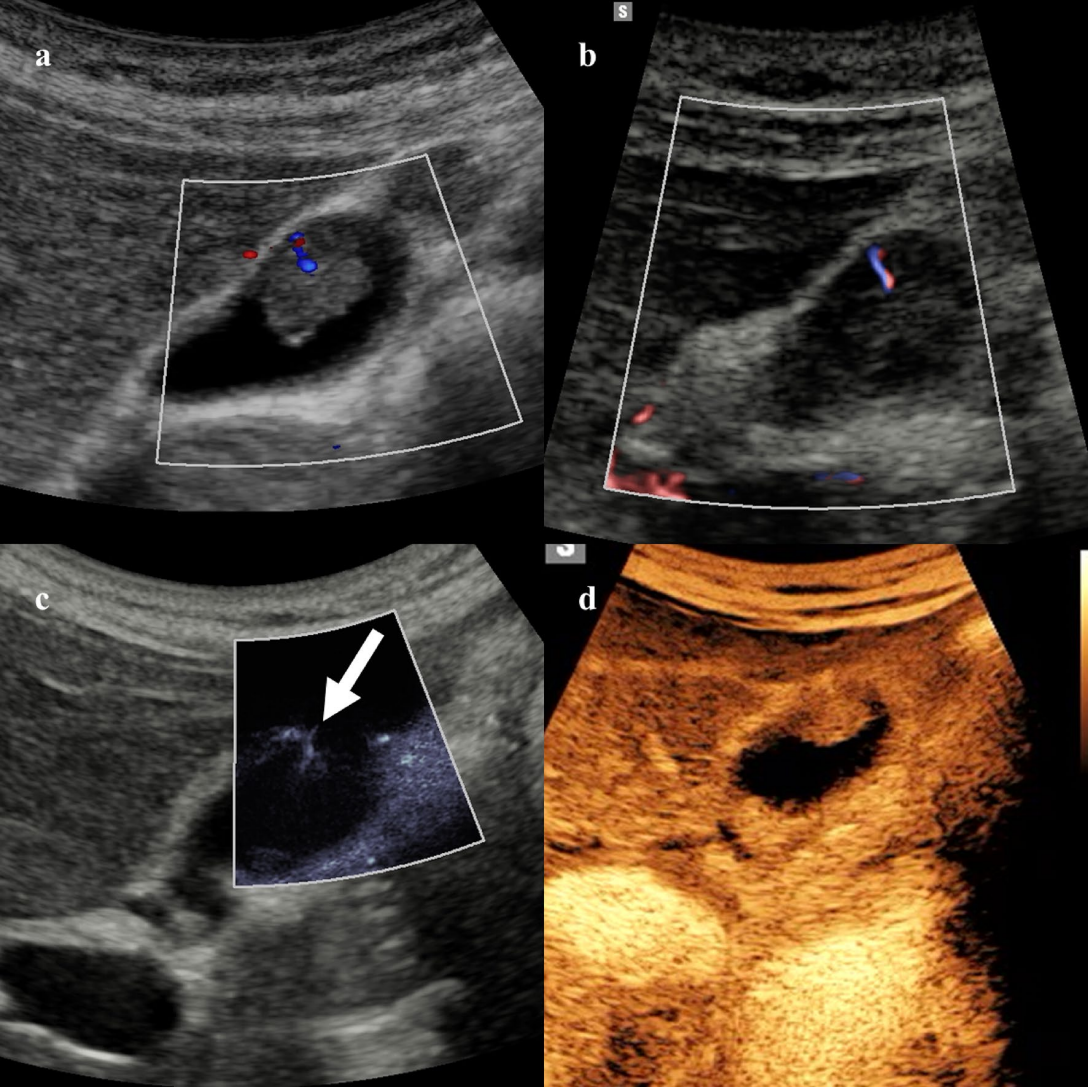
49-year-old woman with incidentally detected 1.2 cm gallbladder polyp. Color (**a**), directional power Doppler (**b**), and microvascular flow imaging (**c**) demonstrate intralesional vascular signal, with branching appearance on MVUS (arrow). The lesion shows homogeneous enhancement on contrast-enhanced ultrasound at 32 s after contrast injection (**d**). This lesion was confirmed as an adenomatous polyp after cholecystectomy

In patients with suspected acute cholecystitis, the quantitative assessment with the superb microvascular imaging allowed to measure the hyperemic changes in the gallbladder bed, improving the diagnosis of acute cholecystitis [34]. Correlation with the severity of inflammation and complications of acute cholecystitis need to be further explored [35, 36]. An initial study including 20 patients also evaluated the feasibility of contrast-enhanced superb microvascular imaging for the vascular evaluation of gallbladder lesions (Fig. 3) demonstrating a significantly higher frequency of tortuous microvessels and abrupt caliber change in malignancies comparted to benign gallbladder lesions [37].

### Pancreas and spleen

Currently, there is a lack of studies supporting the use of MVUS in pancreatic and splenic lesions, with only few published case reports [38, 39]. Particularly, Tokodai et al. [38] applied superb microvascular imaging to monitor splenic vein patency in a patient undergoing pancreatic transplantation. Yamanaka et al. [39] described the microvascular flow findings in two patients with splenic artery pseudoaneurysm. In both cases, MVUS better depicted vascular flow compared to other Doppler techniques. Future studies are needed to explore the potential applications of MVUS in pancreas and spleen.

## Genitourinary applications

### Kidneys

**Fig. 4:**
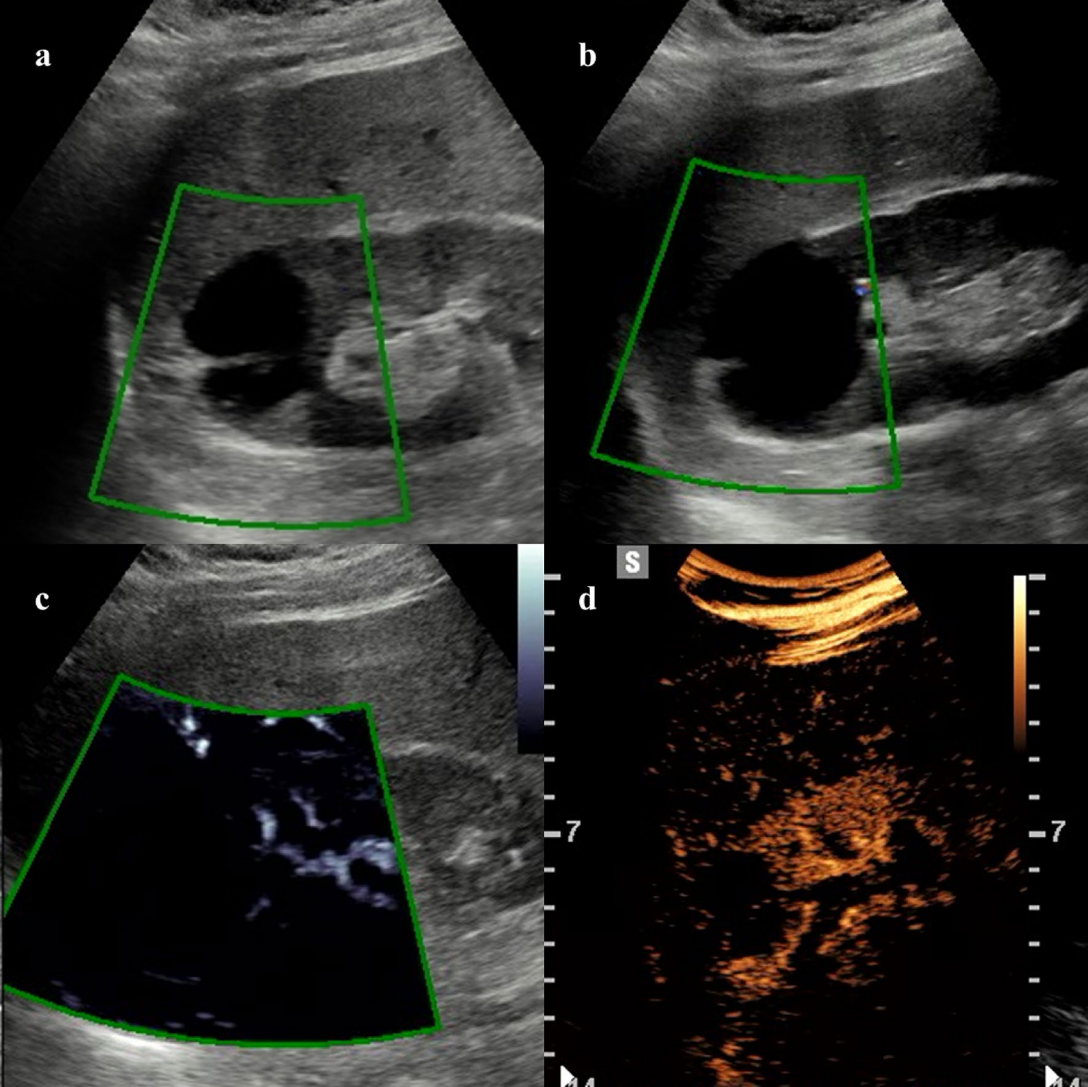
56-year-old man with 5 cm cystic renal lesion detected on computed tomography. Color (**a**), directional power doppler (**b**), and microvascular flow imaging (**c**) do not detect any intracystic vascularization. Contrast-enhanced ultrasound (**d**) shows septa with minimal enhancement at 58 s after contrast injection. The lesion was classified as bosniak IIF

MVUS has the advantage to provide higher sensitivity for the assessment of renal microvasculature compared to conventional Doppler techniques [40]. Improved detection of intralesional vascularization could be particularly helpful in the differential diagnosis of benign and malignant renal tumors. Mao et al. [41] firstly assessed the use of MVUS in 53 patients with solid renal tumors, demonstrating significantly higher vascularity in malignant tumors by using the superb microvascular imaging, with an annular blood flow pattern being more commonly detected in malignant tumors (67% *vs* 9% of benign tumors). Similarly, Leong et al. [42] showed that superb microvascular imaging had the highest sensitivity in detecting tumor vascularity compared to conventional Doppler techniques in 50 indeterminate renal lesions. Chen and colleagues [43] analyzed 63 patients with solid renal tumors with different Doppler techniques. In this study, ring-like blood flow was detected in 37/57 malignant tumors by superb microvascular imaging, which provided a sensitivity of 82.4% and a specificity of 88.8% for the differential diagnosis between benign and malignant lesions [43]. This pattern of vascularization likely correlates with the presence of a pseudocapsule in renal cell carcinoma with fibrotic tissue and capillary vessels. In a large study including 144 solid renal lesions, the intratumoral flow detection rate was reported to be 78.5% with CDI, 88.9% with MVUS, and 93.8% with contrast-enhanced MVUS [44]. Furthermore, superb microvascular imaging demonstrated higher accuracy compared to CDI for the diagnosis of malignancy according to the Bosniak classification in patients with cystic renal lesions (Fig. 4) with improved detection of microvascular flow in cystic septa [45].

MVUS also provided higher detectability of acute pyelonephritis compared to conventional B-mode ultrasound and CDI, with improved detection of hypoperfused cortical areas [46]. Finally, the cortical microvascular flow and vascular index were correlated with the renal function, and lower flow values were associated with the development and severity of chronic kidney disease [47].

In patients receiving kidney transplantation, MVUS showed a decrease of cortical microvasculature with the progressive deterioration of renal function [48]. A recently published study correlated the capsule-to-vessels distance in transplanted kidneys with the chronic allograft damage index, with similar performance of MVUS compared to other Doppler techniques [49].

### Bladder

**Fig. 5:**
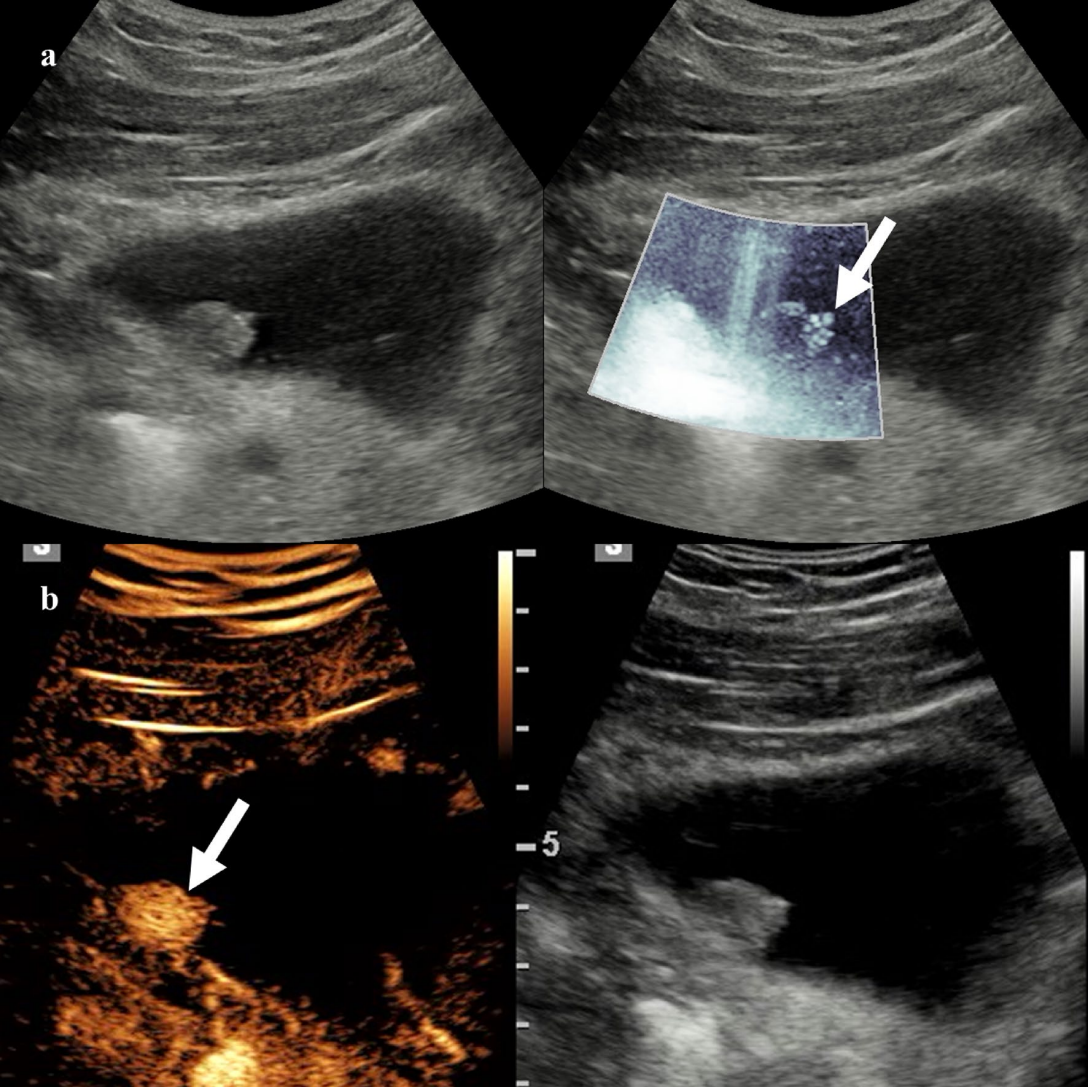
64-year-old man with bladder cancer. Ultrasound examination with microvascular flow imaging (**a**) shows a 2.3 cm lesion of the bladder wall with intralesional vascularization (arrow). The lesion demonstrates strong enhancement on contrast-enhanced ultrasound at 50 s after contrast injection (**b**, arrow)

Few studies investigated the potential applications of MVUS in the urinary bladder (Fig. 5). Ates and coworkers [50] measured the vascular index in the anterior bladder wall to diagnose acute cystitis in pediatric patients with a reported sensitivity of 93% and specificity of 92% for the diagnosis of acute cystitis. Kim et al. [51] applied the superb microvascular imaging in the detection of vesicoureteral reflux, which allowed to demonstrate reversed ureteral jet or renal pelvic swirl sign in 75% of patients.

### Prostate

Transrectal ultrasound-guided biopsy is widely performed to histopathologically diagnose prostate cancer. Zhu et al. [52] analyzed 119 patients who underwent transrectal ultrasound before biopsy. MVUS was able to detect microvascularity in 97.3% of prostatic cancers, and it significantly correlated with the Gleason score [52]. Shen et al. [53] demonstrated that target biopsy guided by the superb microvascular imaging allowed to obtain a higher rate of prostate cancer detection than systematic biopsy. If this data will be confirmed by further studies, MVUS may provide a promising role in guiding target biopsy on transrectal ultrasound.

## Gynecological and obstetric applications

MVUS has been applied to diagnose gynecological and obstetrical conditions in multiple studies. Assessment of vascularization of the ovaries is an important finding in gynecological ultrasound examinations. MVUS demonstrated improved visibility of ovarian vascularity compared to conventional Doppler techniques in healthy patients [54]. In patients with uterine fibroids, Samanci et al. [55] evaluated the role of MVUS for the prediction of response in patients undergoing uterine artery embolization, observing that a higher preoperative vascularization was associated with higher volume reduction.

In pregnant women, MVUS was applied to evaluate placental vascularization and vessels [56–58]. This may be helpful to detect placental abnormalities with higher sensitivity, particularly alterations of placenta attachment or placenta accreta identification [59–61]. Other applications in obstetrics explored the visualization of fetal structures and vasculature such as brain and intra-abdominal vessels [62, 63]. In the initial experience reported by Hata and colleagues [63] superb microvascular imaging was able to visualize abdominal organ microvasculature in the majority of normal fetus at 22–40 weeks of gestation.

## Vascular applications

**Fig. 6:**
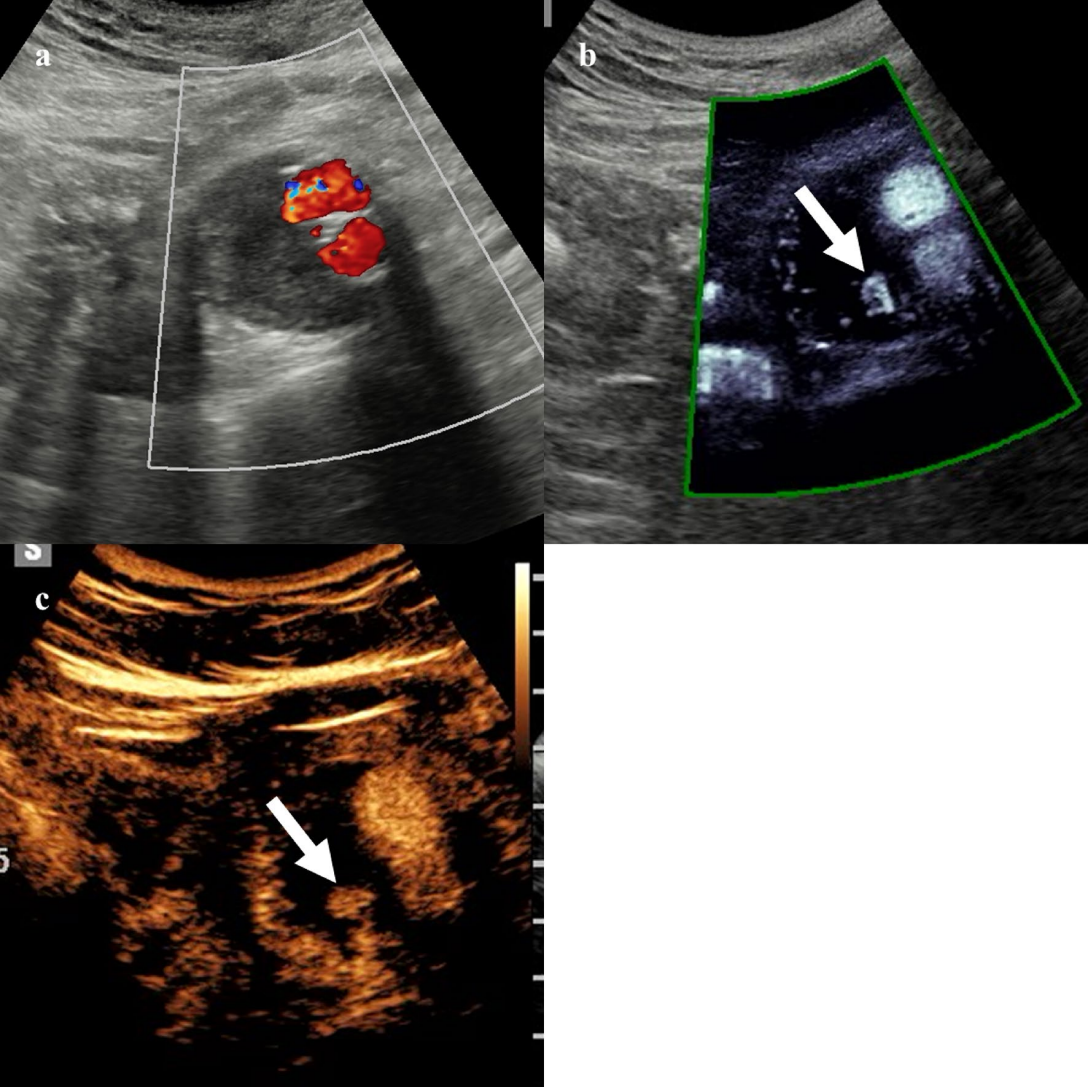
76-year-old man undergoing follow-up examination after endovascular aneurysm repair. Ultrasound examination with color doppler imaging (**a**) shows patency of the graft without signs of endoleak. Microvascular flow imaging (**b**) demonstrates the presence of a peripherally located endoleak (arrow), consistent with type II endoleak from a lumbar artery, which was confirmed on contrast-enhanced ultrasound (**c**, arrow, at 96 s after contrast injection)

In patients undergoing endovascular aneurysm repair (EVAR), ultrasound examination can be performed to monitor the aneurysmal sac size and detect the presence of endoleak, which is characterized by persistent blood flow in the aneurysmal sac [64, 65]. The effectiveness of MVUS for the diagnosis of endoleak (Fig. 6) was explored in recent studies [66–68]. A study by Cantisani et al. [66] firstly demonstrated that MVUS had higher accuracy (63% sensitivity and 96% specificity) for the detection of endoleak compared to CDI in 57 patients treated with EVAR, although the performance was lower compared to CEUS and CT angiography. Gabriel et al. [67] reported the same accuracy of MVUS and CEUS for the detection of endoleak (sensitivity 100%, specificity 93%, accuracy 97%) in 30 patients followed-up after EVAR. Similarly, a recent study by Curti and colleagues [68] reported the same sensitivity (91.5%) and specificity (100%) of MVUS and CEUS for the detection of type II endoleak in 122 patients.

Finally, MVUS has the potential to visualize vascularization in the atherosclerotic plaques, which have an increased risk of complications, but studies on abdominal vessels are still missing [69].

## Conclusion

Microvascular ultrasound imaging improves the detection of vascularization compared to color and power Doppler imaging. The integration of MVUS technology in the new ultrasound equipment provides to radiologists a rapid and versatile tool for the multiparametric ultrasound assessment of various abdominal conditions. In clinical practice, new ultrasound microvascular techniques can be used in conjunct with traditional Doppler imaging to improve the diagnostic performance and detection of vascularity. The vascular patterns detected in hepatic and renal focal lesions have the potential to increase the confidence toward a diagnosis of malignancy or benignity in noncontrast ultrasound examination.
